# Supplementary material for: Chemputer and chemputation—A universal chemical compound synthesis machine
Source: Proc Natl Acad Sci U S A. 2026 Apr 7;123(15):e2511080123. doi: 10.1073/pnas.2511080123 (PMC13080024; doi:10.1073/pnas.2511080123)
Supplement: Supplementary file 1 — Appendix 01 (PDF) [file pnas.2511080123.sapp.pdf]

Supplementary Information:

Chemputer and Chemputation - A Universal Chemical Compound Synthesis Machine

Leroy Cronin*, Sebastian Pagel, and Abhishek Sharma

*School of Chemistry, Advanced Research Centre, University of Glasgow, Glasgow, G11 6EW UK, www.croninlab.com email: lee.cronin@glasgow.ac.uk

1. Simple Example of Chemical Turing Machine

Here, we create the simplest example of an abstract synthetic machine with architecture inspired by the classic Turing Machine. Consider a series of chemical reactions,

$R_1 + R_2 \rightarrow P_1 + P_2$ in solvent S_1 followed by $P_2 + R_3 \rightarrow P_3$ in solvent S_2.

The intermediate products are P_1 and P_2, with the final product being P_3. To perform this synthesis on an abstract synthesis machine, it requires the following process steps:

1. Add reagent R_1

2. Add reagent R_2

3. Add energy to start the reaction, then subtract energy to finish the reaction

4. Add solvent S_1

5. Separate product P_2

6. Add reagent R_3

7. Add energy to start the reaction, then subtract energy to finish the reaction

8. Add solvent S_2

9. Separate product P_3

In principle, we can have an infinite tape of cells (reactors), however, if we know the total number of reagents, in this model, we need twice the number of reactors (including solvents). Each reactor on the tape can have three possible states: 0: empty reactor, 1: filled reactor, 2: active reactor. The filled reactor consists of pre-loaded reagent or solvent for a given reaction, and the active reactor is where the chemical reaction takes place. On top of the tape, there is an active head which can perform mass transfer and hence consists of two possible states: 0: empty (no mass) and 1: full (mass). To keep the construction completely abstract and minimal, we assume the head is capable of adding and subtracting energy locally on the active reactor. As adding energy (electrochemical, photochemical, microwave, heating etc.) and subtract energy (active cooling, dissipating energy to room temperature) are usually analogue quantities, for simplicity, we make two assumptions. First, add energy and subtract energy operations occur in pairs (as an example, heating the active reactor and once the reaction is complete, cooling it

down to room temperature before performing the next step). Second, as both add and subtract energy operations are analogue steps, they could have various different magnitudes and times associated with them. So, the add and subtract energy step could have additional attributes: E_a, τ_a, E_r, τ_r, where E_a and E_r, represent the magnitude of energy flux of the addition and subtraction step and τ_a, and τ_r are the characteristic time scales of the operation. For simplicity, we introduce energy (add and subtract) as a single step on active reactors. If a specific step only requires add energy or only subtract energy, this is equivalent to $\tau_a \rightarrow 0$ or $\tau_r \rightarrow 0$, see Figure S1.

For the given reaction above, with the reagent list (including solvents),

$\{R_1, R_2, S_1, R_3, S_2\}$, the initial state of the tape is:

$\{R_1, E, R_2, E, S_1, E, R_3, E, S_2, E,\}$

where, E are the empty reactors. We initialise the first empty reactor as active,

$\{R_1, A, R_2, E, S_1, E, R_3, E, S_2, E,\}$

which can also be represented as:

{1, 2, 1, 0, 1, 0, 1, 0, 1, 0}

The head is initialised at the leftmost reactor (R_1) and with state 0. The rules are defined as (H is the head state, C is the tape (reactor) state)

if (C==0 && H==0) → C=0, H=0, **move RIGHT**

if (C==1 && H==0) → C=0, H=1, **move RIGHT** [subtract matter]

if (C==0 && H==1) → C=2, H=0, **move LEFT** [add matter]

if (C==1 && H==1) → C=1, H=1, **move RIGHT**

if (C==2 && H==1) → C=2, H=0, **move LEFT** [add matter]

if (C==2 && H==0) → C=0, H=1, **move RIGHT** [add/subtract energy "react" and subtract matter]

These steps are performed until the head reaches the rightmost reactor with C==2, H==0, which is equivalent to the HALTING state, see Figure S1. The state transition diagram for the Turing machine is shown in Figure S2.

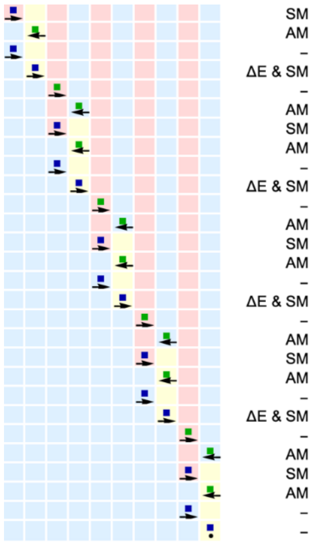

Figure S1: Scheme showing the Turing tape running the reaction with the steps going from top to bottom. The reactor states are shown in large blocks, with red, blue, and yellow shows empty (0), filled (1), and active reactor (2) states. The small square blocks represent the head with dark blue, and green represents empty (0) and full states (1). The circular block at the last step shows the halting state. The arrows show the direction of the head, and the operations performed are shown on the right.

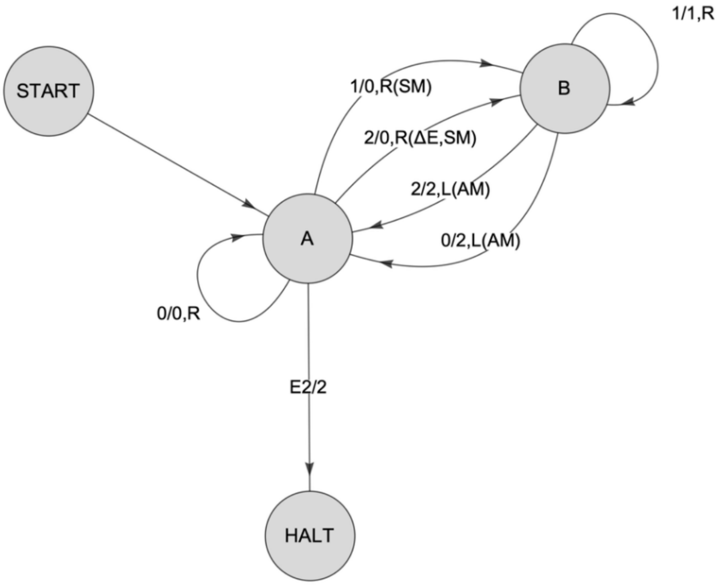

Figure S2: Left: We define the head states as A and B, and the three read/write states on the reactor tape are 0, 1, and 2. The head state A represents when the head is empty (0), and state B represents when the head is loaded with matter (1). As shown previously, the tape states 0, 1, and 2 represent empty reactor, full reactor, and active reactor states. In the figure, the START state represents when the reactor tape is initialised and the head is at the leftmost reactor. Similarly, the HALT state represents when the reaction is finished, which is when the head is empty, and the active reactor is the rightmost reactor. The edge labels represent current tape state/updated tape state and action on the head (R: move right, and L: move left). We also add operations for clarity where AM: add matter, RM: subtract matter, ΔE: add/subtract energy.

2. Classification of XDL operations

Table S1 shows the classification of XDL steps found in the XDL procedures analysed for Figure 7 into four categories: add matter, subtract matter, add energy, and subtract energy. There are some additional steps such as StopPurge, StopHeatChill, which are basic operations for a finite time step and are already incorporated as a part of other XDL steps such as Add, HeatChill. As an example, an abstract add energy step is a combination of HeatChill and StopHeatChill. Figure S3 describes the synthesis procedure classification in XDL in the key primitives, the operations and reaction scheme. Figure S4 shows how the system can be continuously updated and corrected implementing DEC. In the main manuscript, DEC is explicitly indicated in Figures 2 and 3 as acting at reaction execution, work-up, and isolation checkpoints, and as a gatekeeper for CSTM halting conditions.

Table S1 Classification of the unit operations of the Chemputer to the primitives.

**Add Matter**	**Subtract Matter**	**Add Energy**	**Subtract Energy**	**n/a**
Add	Purge	HeatChill(>RT)	HeatChill (<RT)	ResetHandling
AddSolid	Separate	HeatChillToTemp(>RT)	HeatChillToTemp(<RT)	Repeat
Transfer	EvacuateAndRefill	Irradiate	StartHeatChill (<RT)	Wait
Dissolve	Filter	Stir		CleanVessel
	FilterThrough	StartStir		Monitor
	WashSolid	StartHeatChill (>RT)		StopStir
	Dry			StopPurge
	Evaporate			StopHeatChill
	Preparative Chromatography			CSwitchArgon
	Recrystallize			Aysnc
				SwitchVacuum

a)


```
<Synthesis>
  <Hardware>
    <Component
      id="reactor"
      type="reactor" />
    <Component
      id="rotavap"
      type="rotavap" />
    <Component
      id="separator"
      type="separator" />
    <Component
      id="cartridge_MgSO4"
      type="cartridge"
      chemical="MgSO4" />
  </Hardware>

  <Reagents>
    <Reagent
      name="phenylhydrazine"
      role="reagent" />
    <Reagent
      name="acetoveratrone"
      role="reagent" />
    <Reagent
      name="eatons reagent"
      role="reagent" />
    <Reagent
      name="ethanol"
      role="reagent" />
    <Reagent
      name="acetic acid"
      role="reagent" />
    <Reagent
      name="na2co3"
      role="reagent" />
    <Reagent
      name="ch2cl2"
      role="reagent" />
  </Reagents>

  <Procedure>
    <AddSolid
      vessel="reactor"
      reagent="acetoveratrone"
      mass="2.7 g" />
    <Add
      vessel="reactor"
      reagent="phenylhydrazine"
      volume="1.8 mL"
      viscous="True"
      stir="True"
      priming_volume="2 mL" />
    <Add
      vessel="reactor"
      reagent="ethanol"
      volume="10 mL"
      stir="True"
      priming_volume="0" />
    <Add
      vessel="reactor"
      reagent="acetic acid"
      volume="0.3 mL"
      stir="True"
      priming_volume="2 mL" />
    <Add
      vessel="reactor"
      reagent="ethanol"
      volume="20 mL"
      stir="True"
      stir_speed="600 RPM"
      priming_volume="0" />
    <HeatChill
      vessel="reactor"
      temp="70 °C"
      time="3 h"
      stir="True"
      stir_speed="600 RPM" />
    <HeatChillToTemp
      vessel="reactor"
      temp="55 °C"
      active="False"
      continue_heatchill="False"
      stir="True" />
    <Evaporate
      vessel="reactor"
      temp="60 °C"
      pressure="20 mbar" />
    <HeatChillToTemp
      vessel="reactor"
      temp="30 °C"
      active="False"
      continue_heatchill="False"
      stir="True" />
    <Add
      vessel="reactor"
      reagent="eatons reagent"
      volume="15 mL"
      stir="True"
      stir_speed="600 RPM"
      priming_volume="2 mL" />
    <HeatChill
      vessel="reactor"
      temp="60 °C"
      time="48 h"
      stir="True"
      stir_speed="600 RPM" />
    <HeatChillToTemp
      vessel="reactor"
      temp="30 °C"
      active="False"
      continue_heatchill="False"
      stir="True" />
    <Add
      vessel="reactor"
      reagent="na2co3"
      volume="20 mL"
      stir="True"
      priming_volume="0" />
    <Repeat
      repeats="2">
      <Add
        vessel="reactor"
        reagent="ch2cl2"
        volume="20 mL"
        stir="True"
        priming_volume="0" />
      <Add
        vessel="reactor"
        reagent="na2co3"
        volume="20 mL"
        stir="True"
        priming_volume="0" />
      <StopStir
        vessel="reactor" />
      <Transfer
        from_vessel="reactor"
        to_vessel="separator"
        volume="all" />
    </Repeat>
    <Separate
      purpose="extract"
      from_vessel="separator"
      separation_vessel="separator"
      to_vessel="separator"
      product_phase="bottom"
      solvent="ch2cl2"
      solvent_volume="40 mL"
      repeats="3"
      remove_dead_volume="False" />
    <Transfer
      from_vessel="separator"
      to_vessel="rotavap"
      volume="all"
      through="mgso4"
      move_speed="10.0" />
    <Transfer
      from_vessel="separator"
      to_vessel="rotavap"
      volume="30 mL"
      through="mgso4" />
    <Evaporate
      vessel="rotavap"
      temp="40 °C"
      pressure="50 mbar"
      time="60 min"
      mode="auto" />
  </Procedure>
</Synthesis>
```


Initial state | Add Matter | Add Energy | Subtract Matter

b)

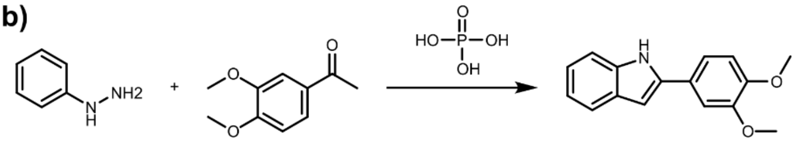

Figure S3: a) Classification of synthesis steps represented as a XDL procedure using the Chemputation primitives. **b)** Reaction scheme of the reaction procedure described in a).

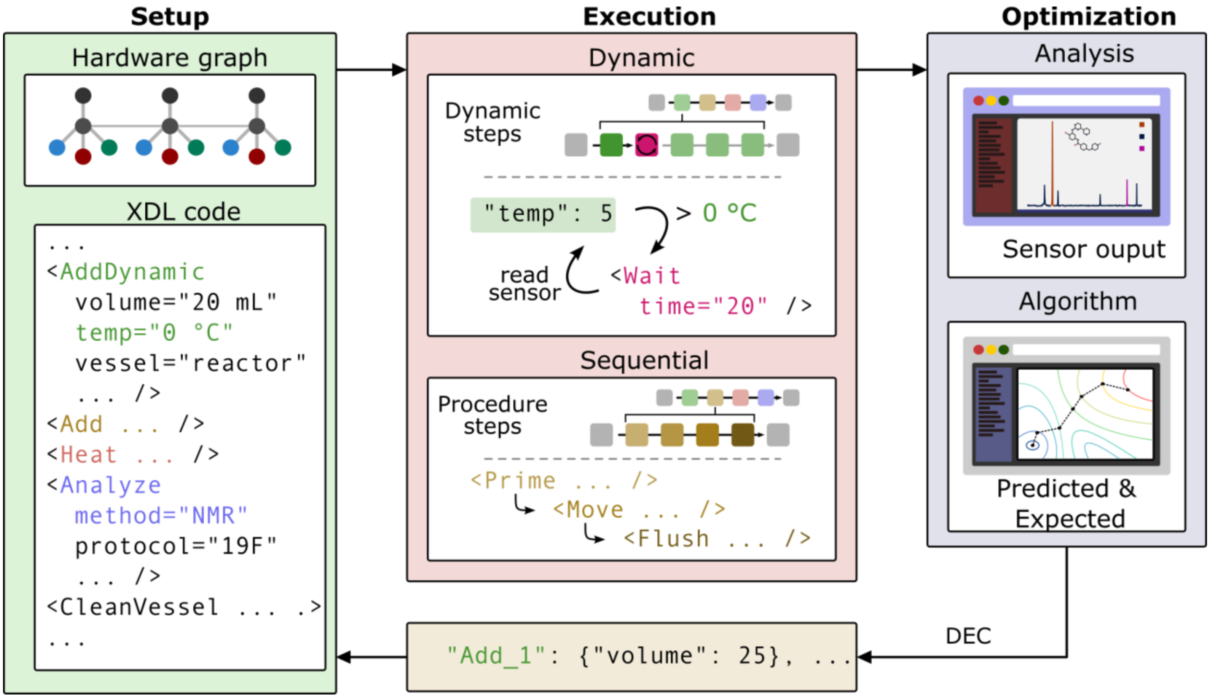

Figure S4: Overview of i) setup of the Chemputation in terms of the graph and the initial XDL code; ii) The execution using dynamic steps; iii) the parameter update for the optimization which together corresponds to the DEC process outlined. Adapted from Leonov et. al., ref 46.

3. Description of experimental protocols using XDL

All the details of the reactions, their scale, and yield are given below in Table S2. The experimental protocols are available as XDLs, together with the corresponding analytical data, and can be accessed at Rohrbach et al. (ref 8).

Table S2. List of the reactions, reaction scale, yield and scheme on the following pages. Missing values indicate that the XDL protocols were manually validated and simulated without experimental execution. All reaction images were created with RDKitv2025.9.3:

ID	Step	Yield	Scale	Reaction
CHEMIFY-0001	Step 1	98 %	8.8 mmol	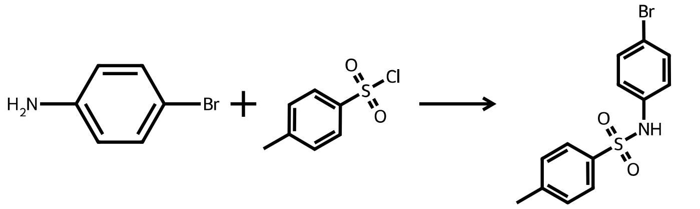
CHEMIFY-0002	Step 1	56 %	20.0 mmol	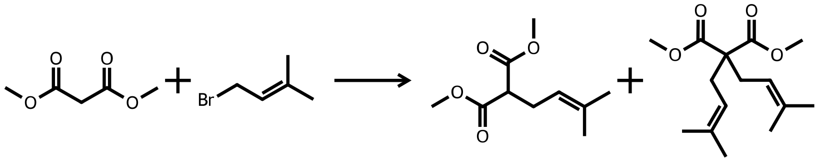
CHEMIFY-0004	Step 1	84 %	10.0 mmol	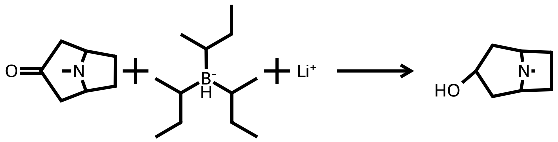

ID	Step	Yield	Scale	Reaction
CHEMIFY-0005	Step 1	37 %	13.0 mmol	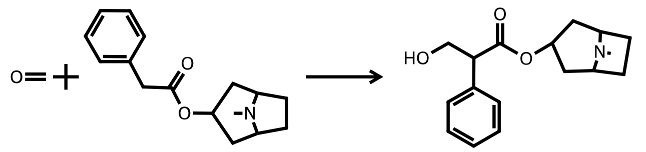
CHEMIFY-0006	Step 1	65 %	20.0 mmol	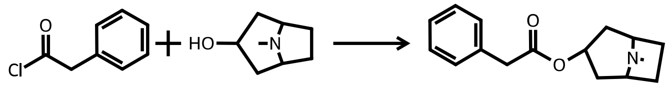
CHEMIFY-0010	Step 1	43 %	10.0 mmol	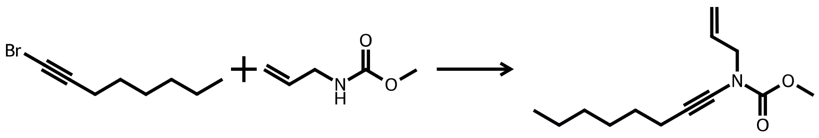

ID	Step	Yield	Scale	Reaction
CHEMIFY-0011	Step 1	94 %	5.0 mmol	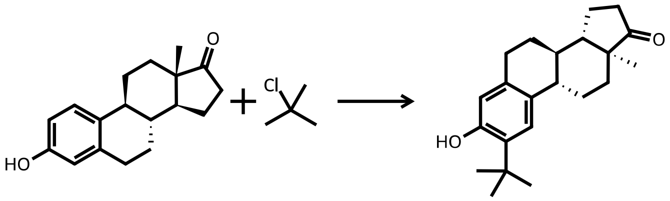
CHEMIFY-0012	Step 1	92 %	2.0 mmol	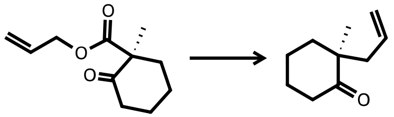
CHEMIFY-0013	Step 1	62 %	15.0 mmol	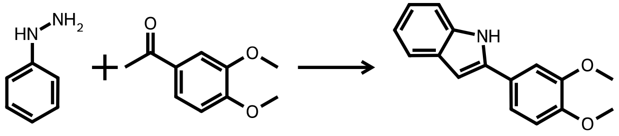

ID	Step	Yield	Scale	Reaction
CHEMIFY-0014	Step 1	60 %	25.1 mmol	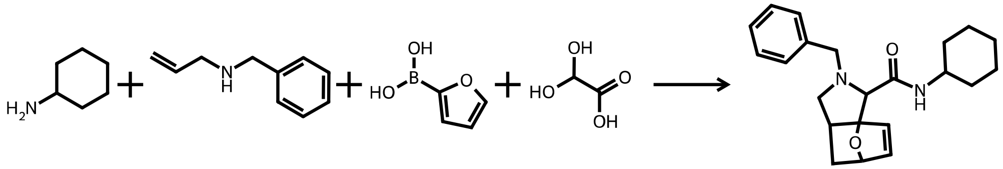
CHEMIFY-0015	Step 1	56 %	0.5 mmol	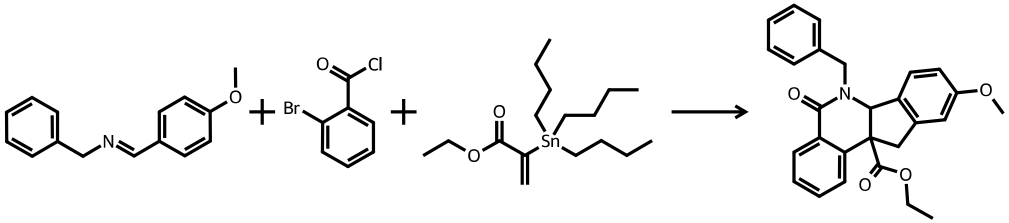
CHEMIFY-0016	Step 1	64 %	12.0 mmol	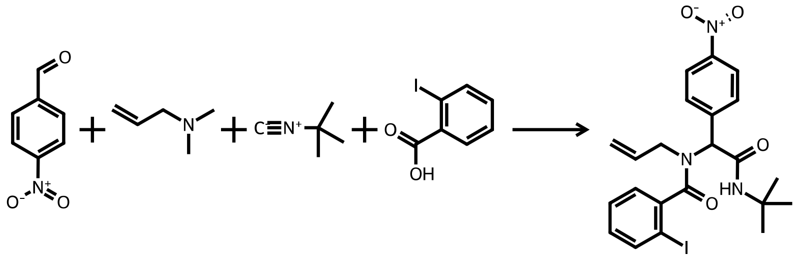

ID	Step	Yield	Scale	Reaction
CHEMIFY-0017	Step 1	86 %	5.0 mmol	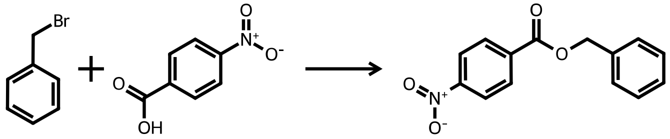
CHEMIFY-0018	Step 1	36 %	100.0 mmol	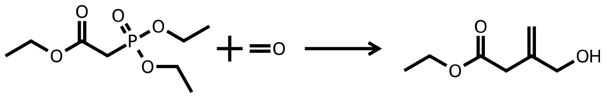
CHEMIFY-0020	Step 1		4.0 mmol	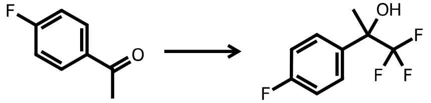

ID	Step	Yield	Scale	Reaction
CHEMIFY-0021	Step 1	68.8 %	2.64 mmol	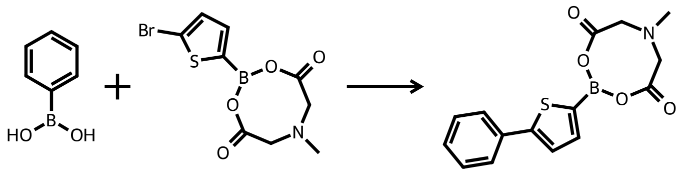
CHEMIFY-0022	Step 1	32 %	1.86 mmol	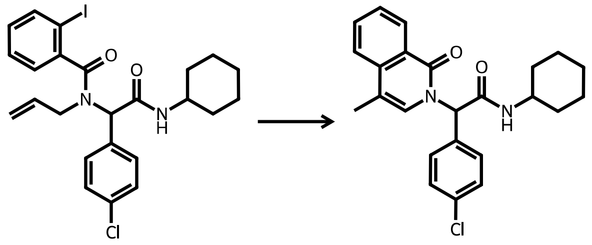
CHEMIFY-0023	Step 1	35 %	6.0 mmol	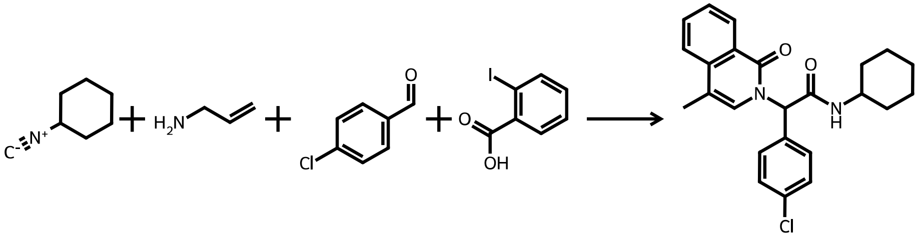

ID	Step	Yield	Scale	Reaction
CHEMIFY-0024	Step 1	65 %	3.10 mmol	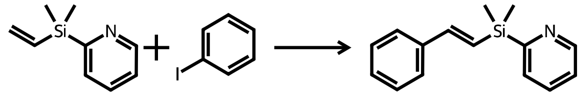
CHEMIFY-0025	Step 1	74 %	3.10 mmol	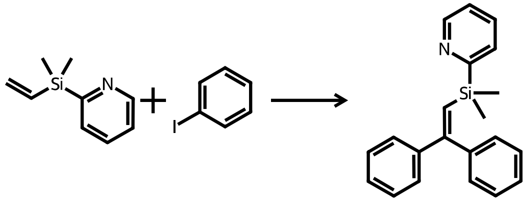
CHEMIFY-0026	Step 1	61 %	5.02 mmol	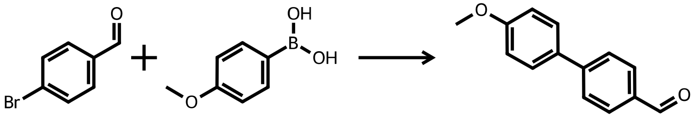

ID	Step	Yield	Scale	Reaction
CHEMIFY-0027	Step 1	84 %	5.0 mmol	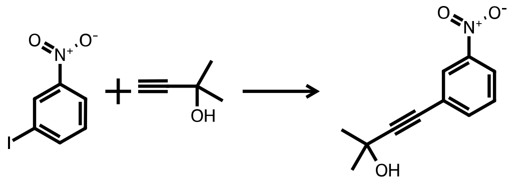
CHEMIFY-0028	Step 1	66 %	50.0 mmol	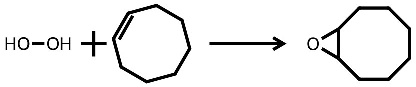
CHEMIFY-0029	Step 1	62 %	25.0 mmol	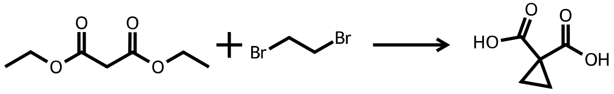

ID	Step	Yield	Scale	Reaction
CHEMIFY-0030	Step 1	88 %	5.0 mmol	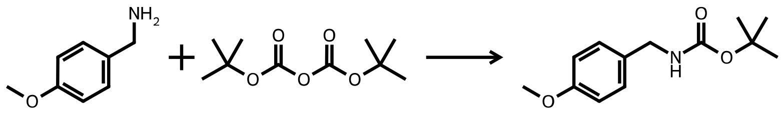
CHEMIFY-0032	Step 1	36 %	40.0 mmol	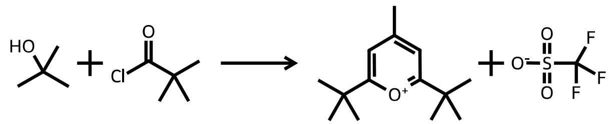
CHEMIFY-0033	Step 1	59 %	33.8 mmol	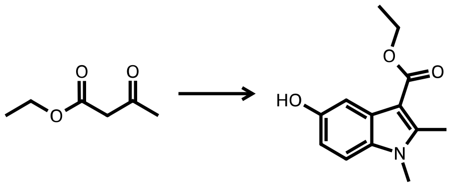

ID	Step	Yield	Scale	Reaction
CHEMIFY-0034	Step 1	81 %	90.5 mmol	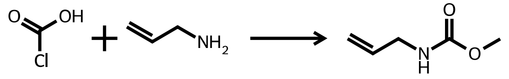
CHEMIFY-0035	Step 1	91 %	10.0 mmol	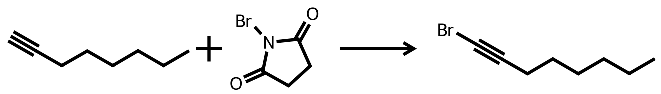
CHEMIFY-0037	Step 1	74 %	9.0 mmol	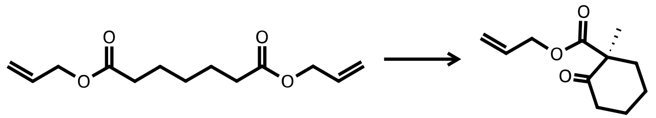

ID	Step	Yield	Scale	Reaction
CHEMIFY-0038	Step 1	52 %	45.0 mmol	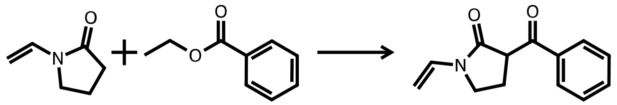
CHEMIFY-0040	Step 1	36 %	33.3 mmol	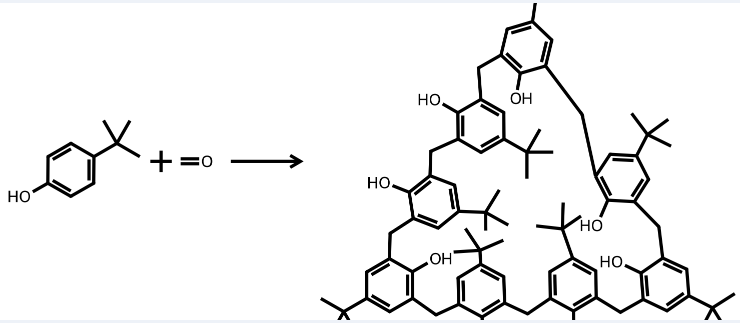
CHEMIFY-0041	Step 1	65 %	25.6 mmol	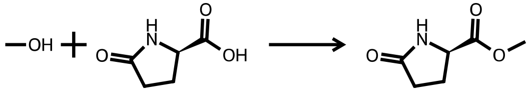

ID	Step	Yield	Scale	Reaction
CHEMIFY-0042	Step 1	58 %	0.77 mmol	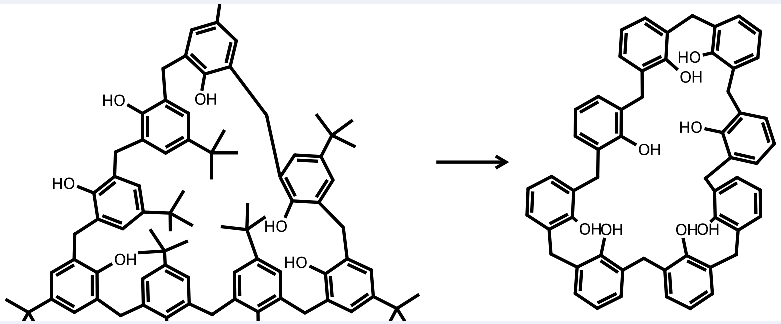
CHEMIFY-0043	Step 1	76 %	100.0 mmol	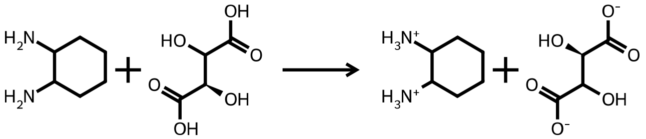
CHEMIFY-0044	Step 1	85 %	11.2 mmol	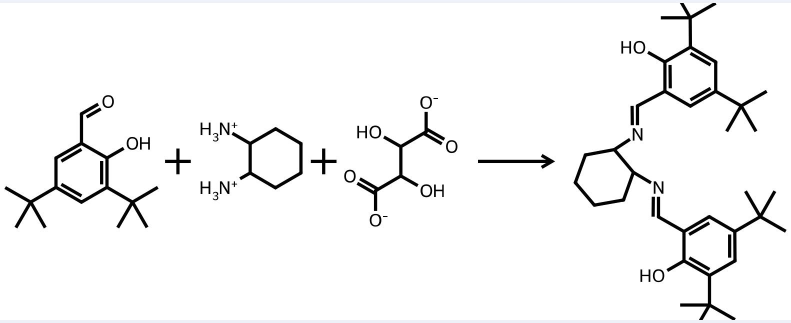

ID	Step	Yield	Scale	Reaction
CHEMIFY-0045	Step 1	70 %	1.5 mmol	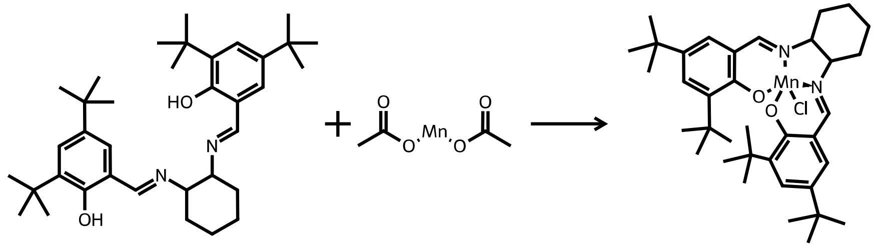
CHEMIFY-0046	Step 1	99 %	15.0 mmol	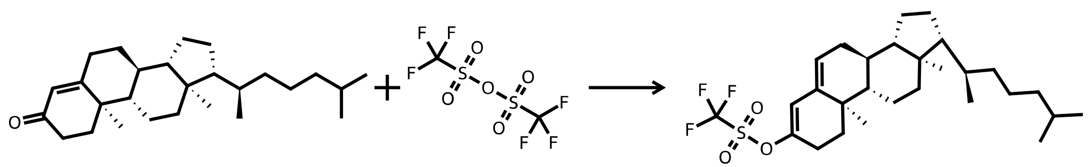
CHEMIFY-0047	Step 1	71 %	5.00 mmol	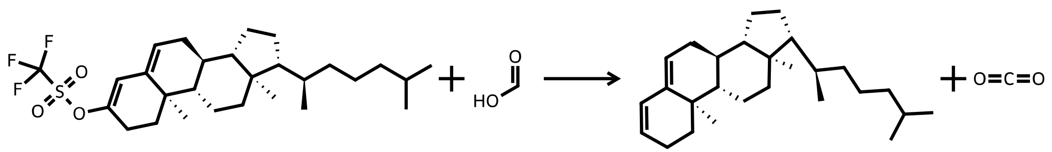

ID	Step	Yield	Scale	Reaction
CHEMIFY-0048	Step 1	40 %	34.9 mmol	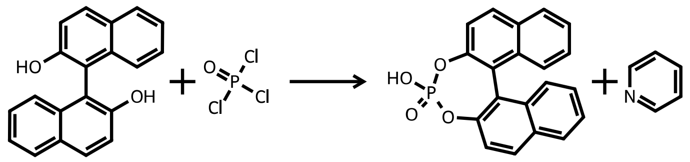
CHEMIFY-0049	Step 1		20.0 mmol	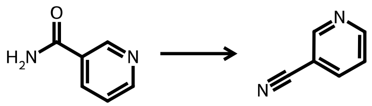
CHEMIFY-0050	Step 1	65 %	1.35 mmol	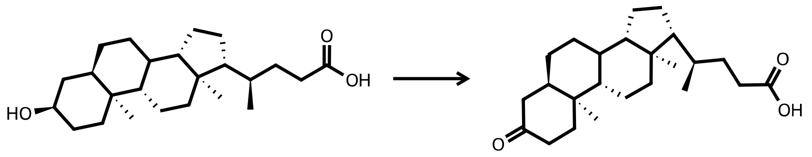

ID	Step	Yield	Scale	Reaction
CHEMIFY-0051	Step 1	76 %	33.0 mmol	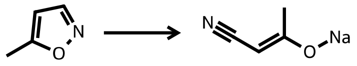
CHEMIFY-0052	Step 1	53 %	10.1 mmol	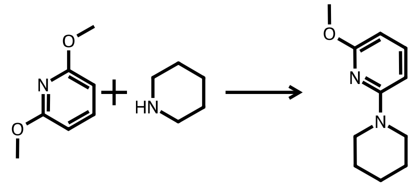
CHEMIFY-0053	Step 1	75 %	5.0 mmol	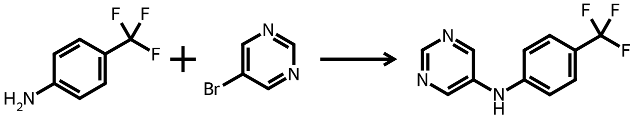

ID	Step	Yield	Scale	Reaction
CHEMIFY-0054	Step 1	56 %	12.0 mmol	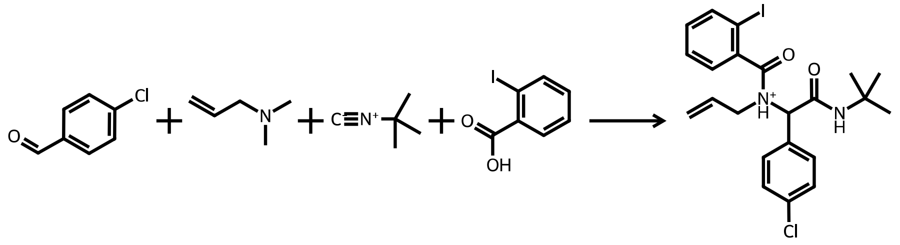
CHEMIFY-0055	Step 1	80 %	12.0 mmol	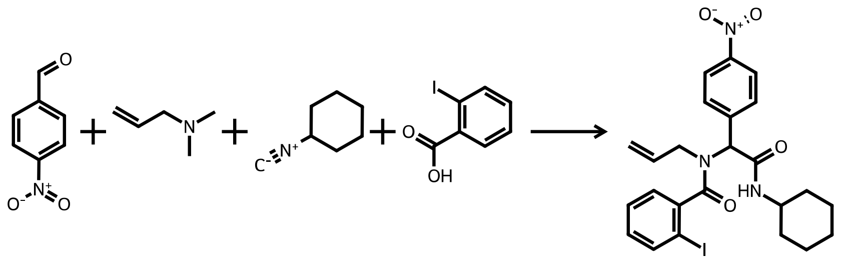
CHEMIFY-0056	Step 1	78 %	12.0 mmol	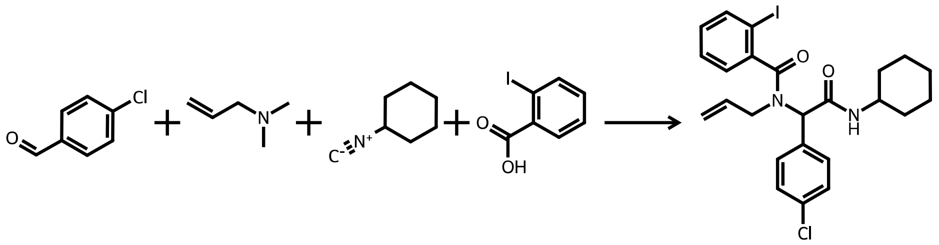

ID	Step	Yield	Scale	Reaction
CHEMIFY-0057	Step 1	31 %	20.0 mmol	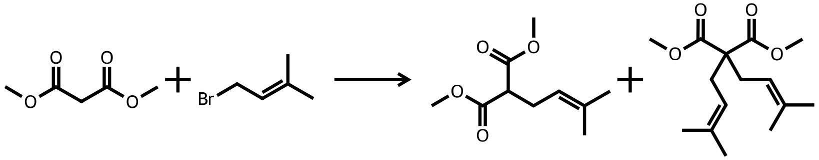
CHEMIFY-0075	Step 1	56 %	20 mmol	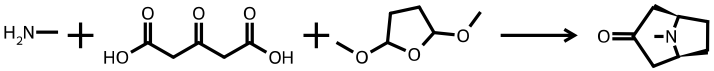
CHEMIFY-0077	Step 1	33 %	30 mmol	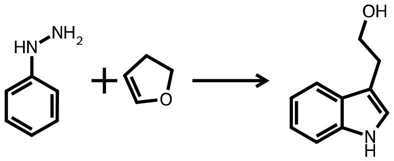

ID	Step	Yield	Scale	Reaction
CHEMIFY-0078	Step 1	64 %	40 mmol	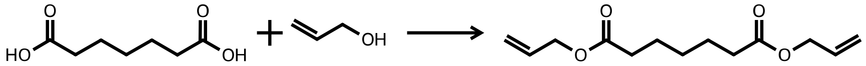
CHEMIFY-0079	Step 1	38 %	10 mmol	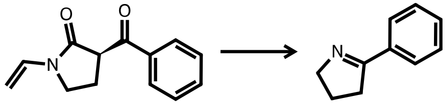
CHEMIFY-0080	Step 1			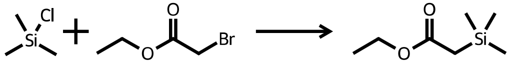

ID	Step	Yield	Scale	Reaction
CHEMIFY-0081	Step 1			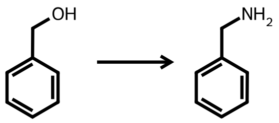
CHEMIFY-0082	Step 1			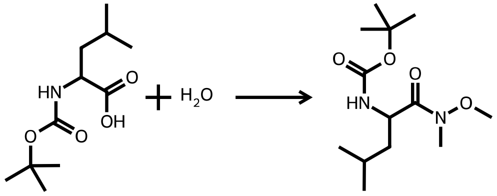
CHEMIFY-0084	Step 1			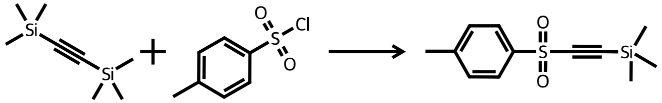

ID	Step	Yield	Scale	Reaction
CHEMIFY-0085	Step 1			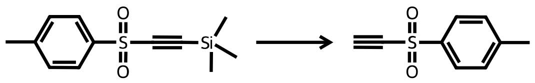
CHEMIFY-0086	Step 1			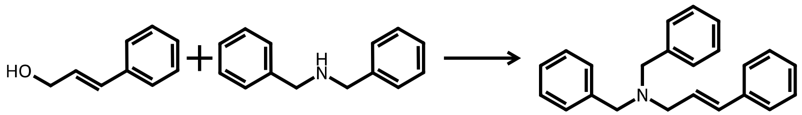
CHEMIFY-0087	Step 1			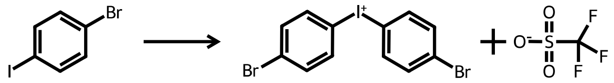

ID	Step	Yield	Scale	Reaction
CHEMIFY-0088	Step 1			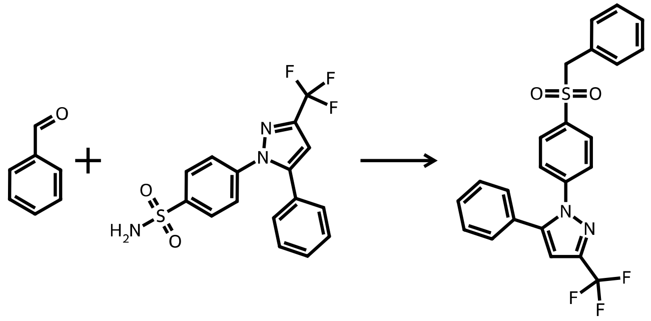
CHEMIFY-0089	Step 1			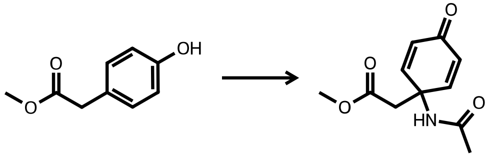
CHEMIFY-0090	Step 1			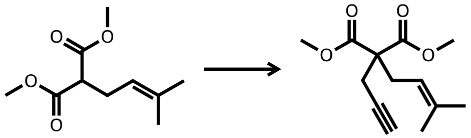

ID	Step	Yield	Scale	Reaction
CHEMIFY-0091	Step 1			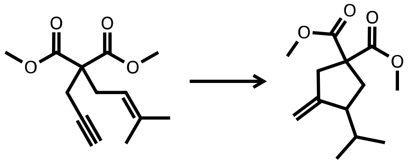
CHEMIFY-0092	Step 1			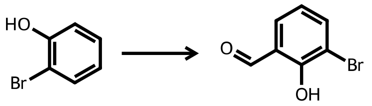
CHEMIFY-0093	Step 1			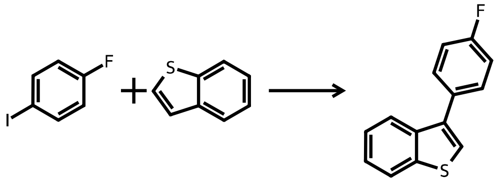

ID	Step	Yield	Scale	Reaction
CHEMIFY-0094	Step 1			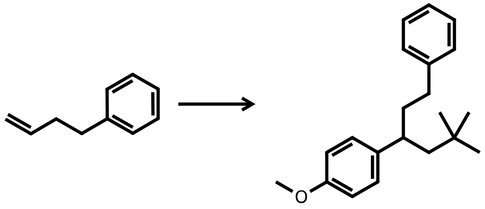
CHEMIFY-0095	Step 1			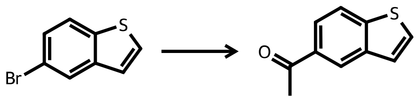
CHEMIFY-0096	Step 1			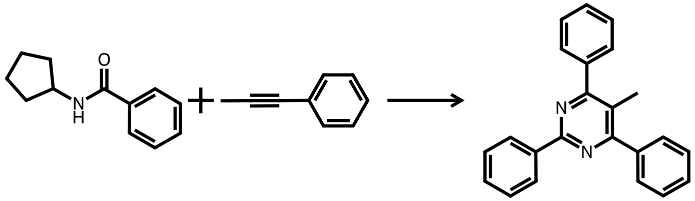

ID	Step	Yield	Scale	Reaction
CHEMIFY-0097	Step 1			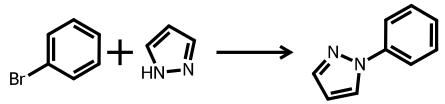
CHEMIFY-0098	Step 1			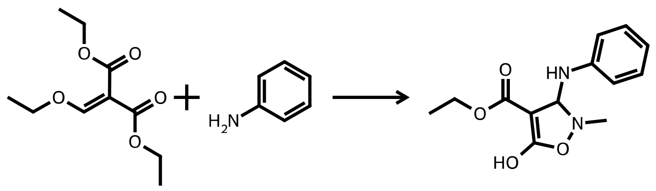
CHEMIFY-0099	Step 1			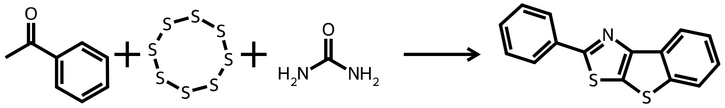

ID	Step	Yield	Scale	Reaction
CHEMIFY-0100	Step 1			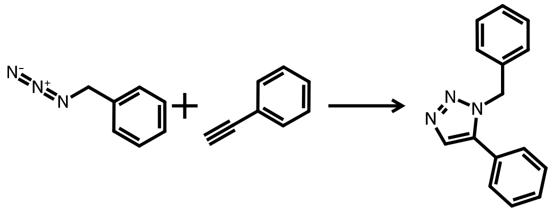
CHEMIFY-0101	Step 1			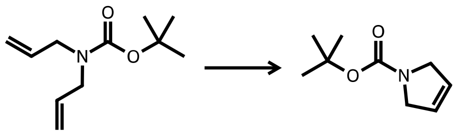
CHEMIFY-0102	Step 1			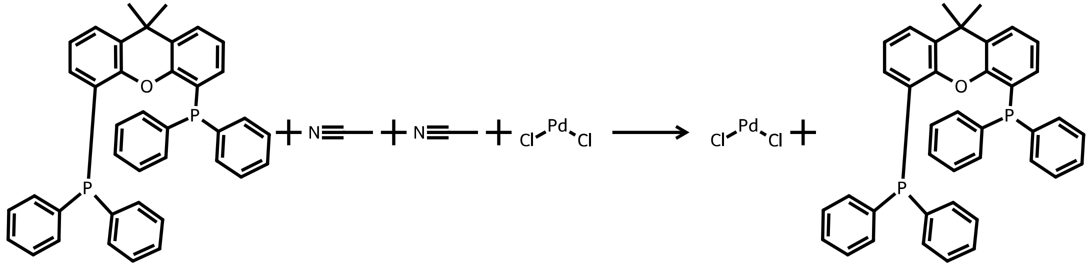

ID	Step	Yield	Scale	Reaction
CHEMIFY-0103	Step 1			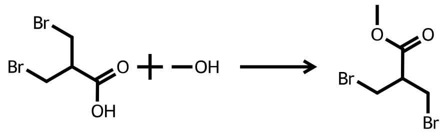
CHEMIFY-0104	Step 1			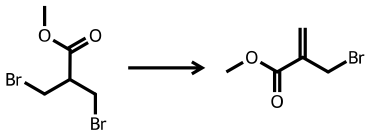
CHEMIFY-0105	Step 1			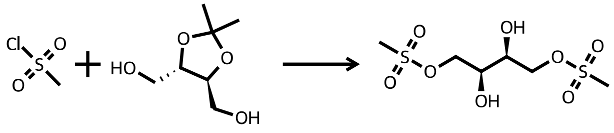

ID	Step	Yield	Scale	Reaction
CHEMIFY-0106	Step 1			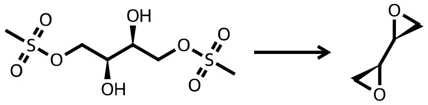
CHEMIFY-0107	Step 1			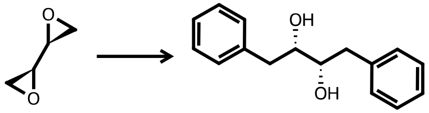
CHEMIFY-0108	Step 1			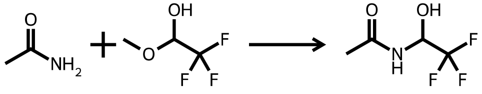

ID	Step	Yield	Scale	Reaction
CHEMIFY-0109	Step 1			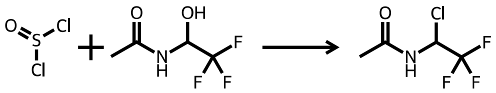
CHEMIFY-0110	Step 1			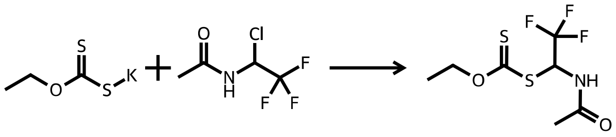
CHEMIFY-0111	Step 1			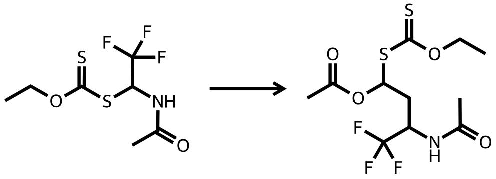

ID	Step	Yield	Scale	Reaction
CHEMIFY-0112	Step 1			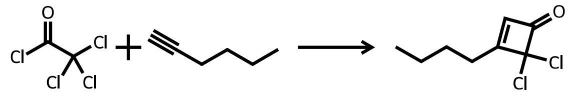
CHEMIFY-0113	Step 1			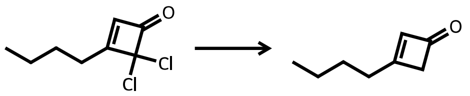
CHEMIFY-0114	Step 1			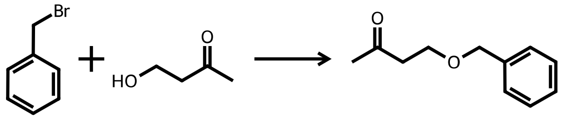

ID	Step	Yield	Scale	Reaction
CHEMIFY-0115	Step 1			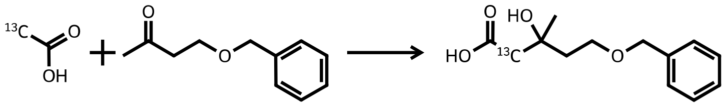
CHEMIFY-0116	Step 1			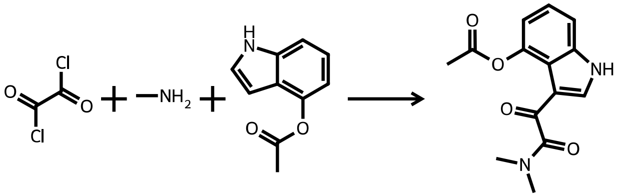
CHEMIFY-0117	Step 1			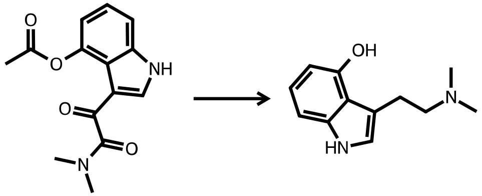

ID	Step	Yield	Scale	Reaction
CHEMIFY-0118	Step 1			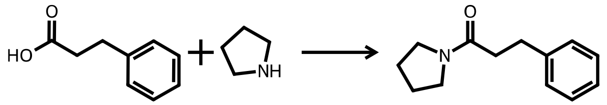
CHEMIFY-0119	Step 1			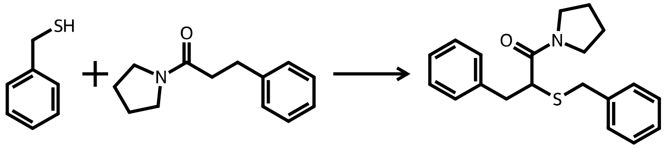
CHEMIFY-0120	Step 1			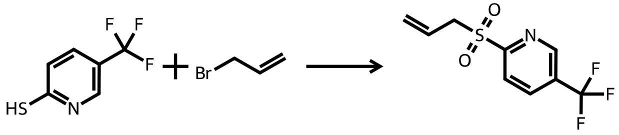

ID	Step	Yield	Scale	Reaction
CHEMIFY-0121	Step 1			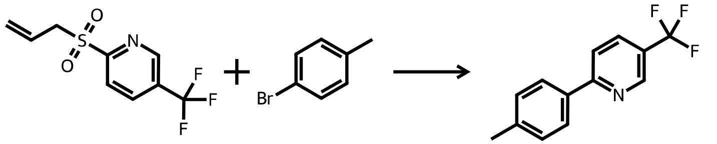
CHEMIFY-0122	Step 1			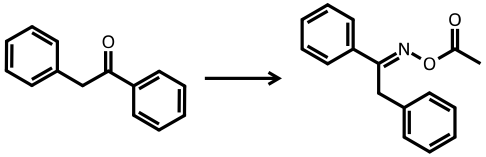
CHEMIFY-0123	Step 1			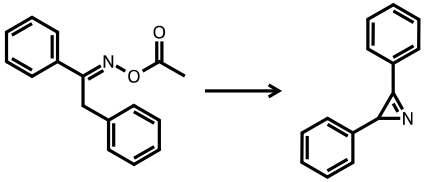

ID	Step	Yield	Scale	Reaction
CHEMIFY-0124	Step 1			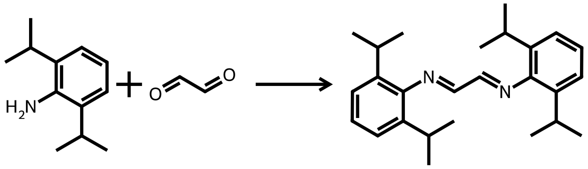
CHEMIFY-0125	Step 1			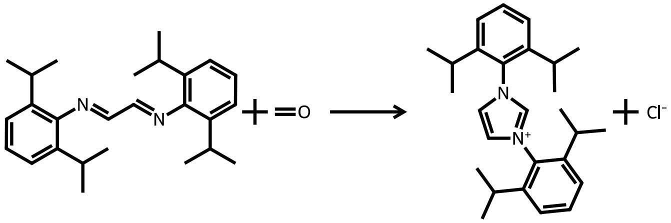
CHEMIFY-0126	Step 1			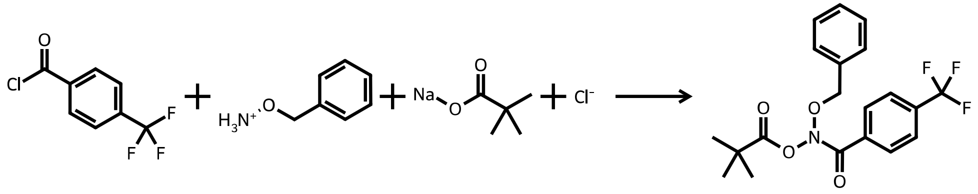

ID	Step	Yield	Scale	Reaction
CHEMIFY-0127	Step 1			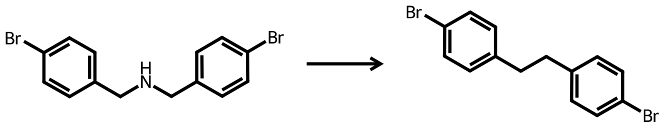
TwoStep-1 (0034+0010)	Step 1	81 %, 43 %	90.5 mmol, 10.0 mmol	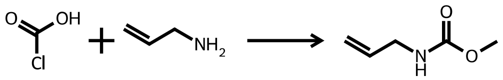
	Step 2			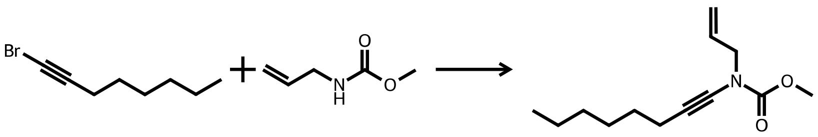

ID	Step	Yield	Scale	Reaction
TwoStep-2 (0035+0010)	Step 1	91 %, 43 %	10.0 mmol, 10.0 mmol	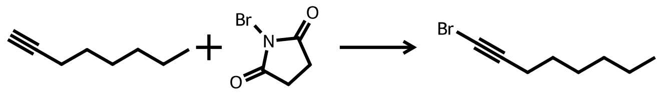
	Step 2			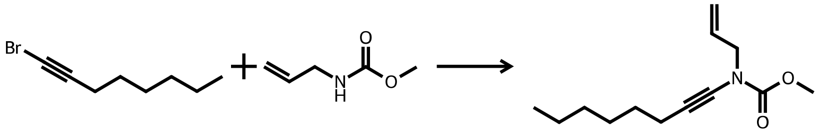
TwoStep-3 (0078+0037)	Step 1	64 %, 74 %	40 mmol, 9.0 mmol	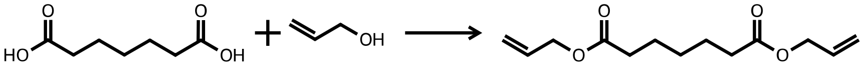

ID	Step	Yield	Scale	Reaction
	Step 2			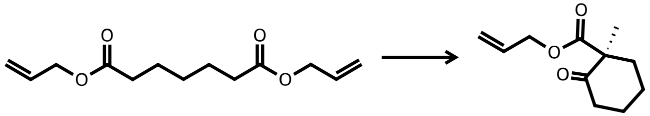
TwoStep-4 (0037+0012)	Step 1	74 %, 92 %	9.0 mmol, 2.0 mmol	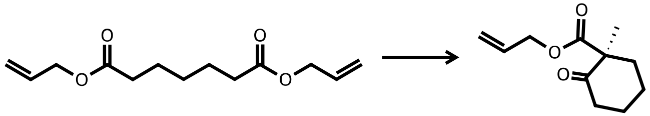
	Step 2			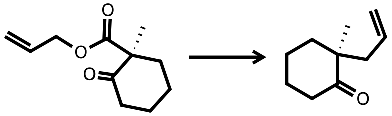

ID	Step	Yield	Scale	Reaction
TwoStep-5 (0075+0004)	Step 1	56 %, 84 %	20 mmol, 10.0 mmol	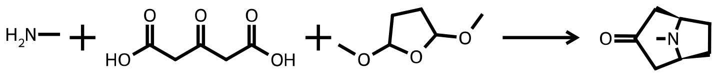
	Step 2			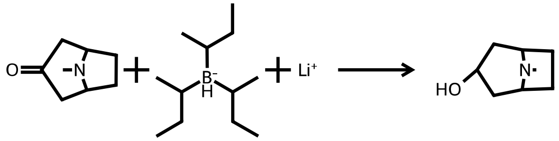
TwoStep-6 (0004+0005)	Step 1	84 %, 37 %	10.0 mmol, 13.0 mmol	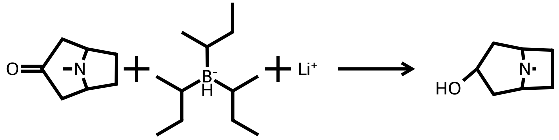

ID	Step	Yield	Scale	Reaction
	Step 2			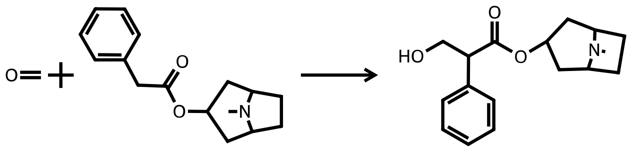
TwoStep-7 (0056+0022)	Step 1	78 %, 32 %	12.0 mmol, 1.86 mmol	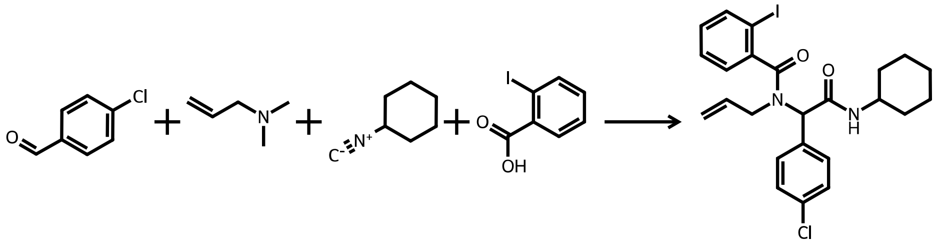
	Step 2			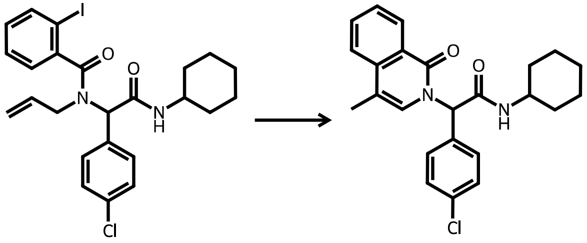

ID	Step	Yield	Scale	Reaction
TwoStep-8 (0056+0023)	Step 1	78 %, 35 %	12.0 mmol, 6.0 mmol	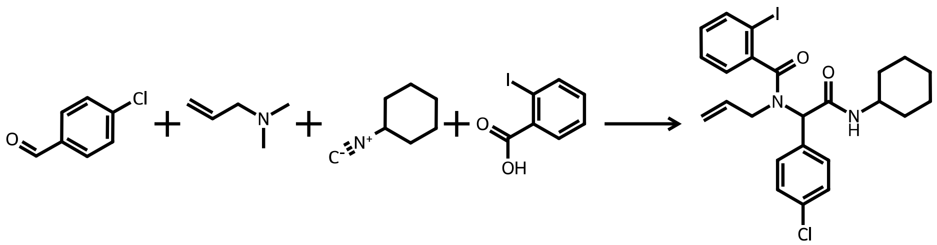
	Step 2			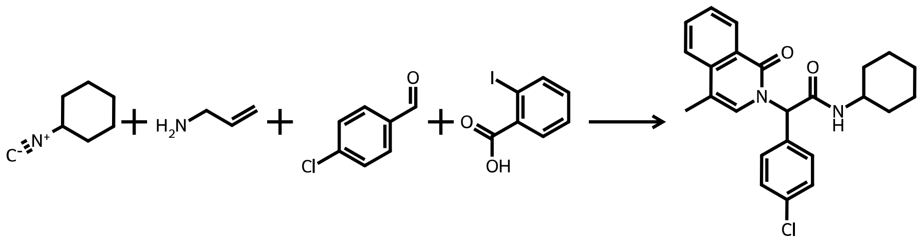
TwoStep-9 (0038+0079)	Step 1	52 %, 38 %	45.0 mmol, 10 mmol	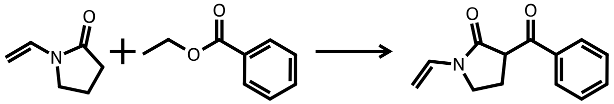

ID	Step	Yield	Scale	Reaction
	Step 2			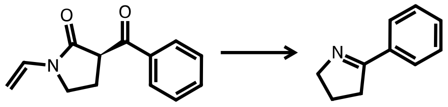
TwoStep-10 (0040+0042)	Step 1	36 %, 58 %	33.3 mmol, 0.77 mmol	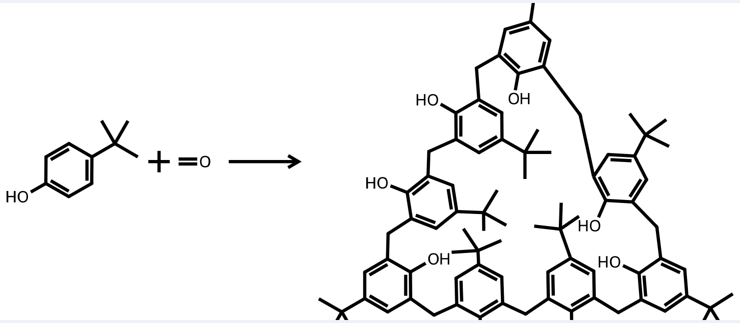
	Step 2			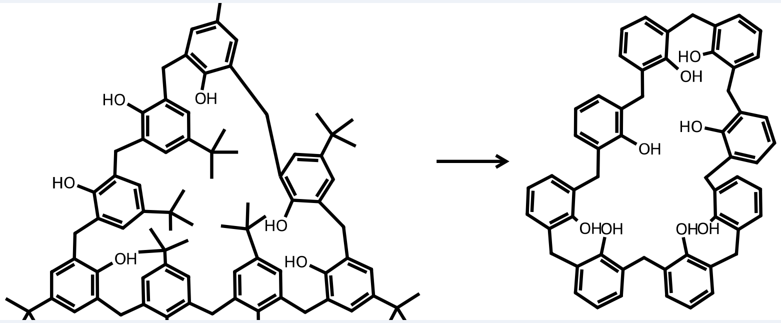

ID	Step	Yield	Scale	Reaction
TwoStep-11 (0043+0044)	Step 1	76 %, 85 %	100.0 mmol, 11.2 mmol	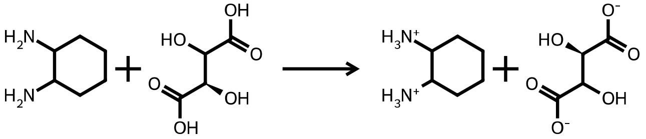
	Step 2			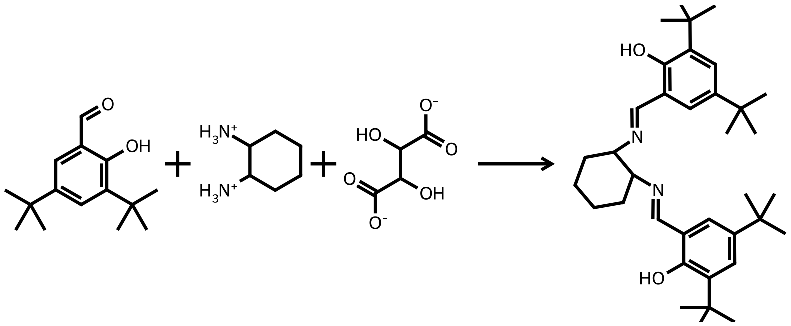
TwoStep-12 (0044+0045)	Step 1	85 %, 70 %	11.2 mmol, 1.5 mmol	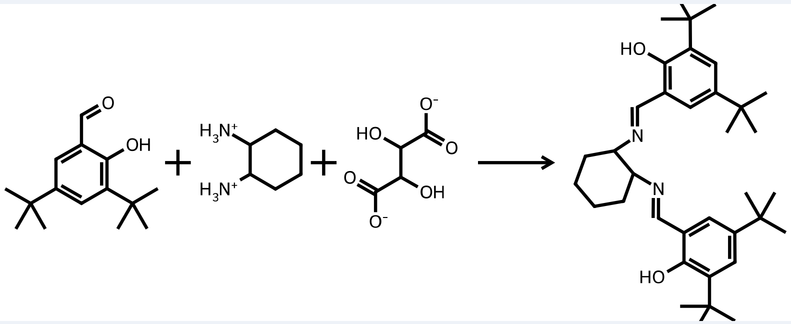

ID	Step	Yield	Scale	Reaction
	Step 2			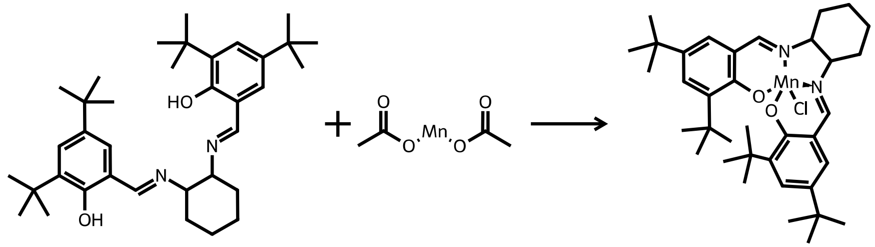
TwoStep-13 (0046+0047)	Step 1	99 %, 71 %	15.0 mmol, 5.00 mmol	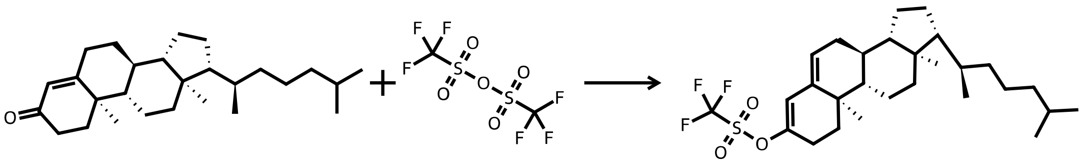
	Step 2			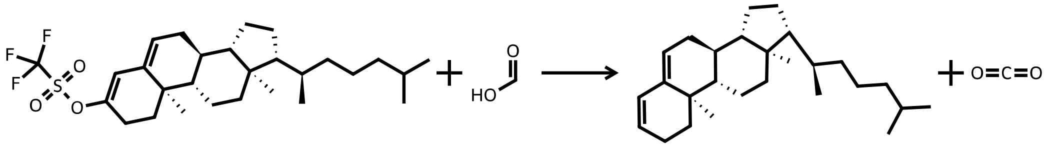

ID	Step	Yield	Scale	Reaction
ThreeStep-1 (0034+0035+0010)	Step 1	81 %, 91 %, 43 %	90.5 mmol, 10.0 mmol, 10.0 mmol	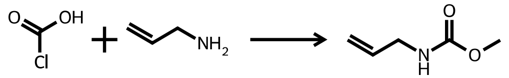
	Step 2			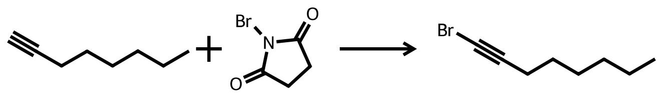
	Step 3			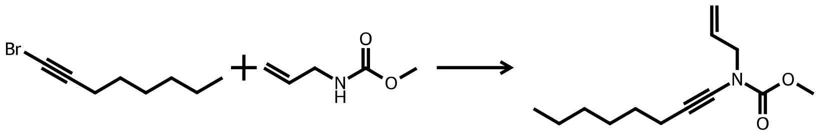

ID	Step	Yield	Scale	Reaction
ThreeStep-2 (0078+0037+0012)	Step 1	64 %, 74 %, 92 %	40 mmol, 9.0 mmol, 2.0 mmol	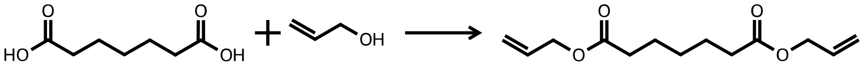
	Step 2			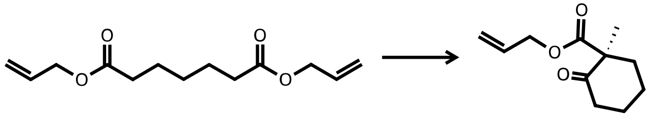
	Step 3			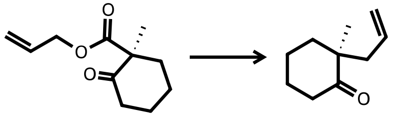

ID	Step	Yield	Scale	Reaction
ThreeStep-3 (0075+0004+0005)	Step 1	56 %, 84 %, 37 %	20 mmol, 10.0 mmol, 13.0 mmol	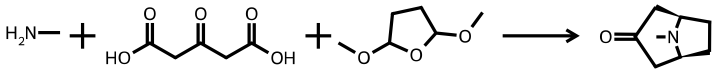
	Step 2			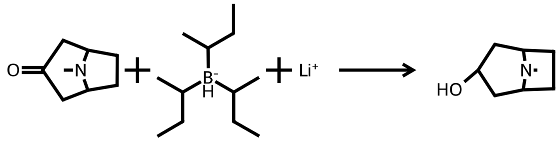
	Step 3			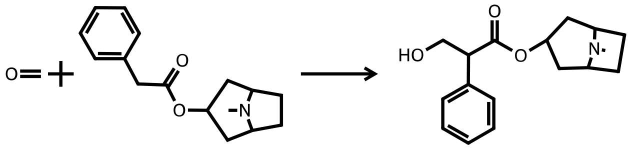

ID	Step	Yield	Scale	Reaction
ThreeStep-4 (0043+0044+0045)	Step 1	76 %, 85 %, 70 %	100.0 mmol, 11.2 mmol, 1.5 mmol	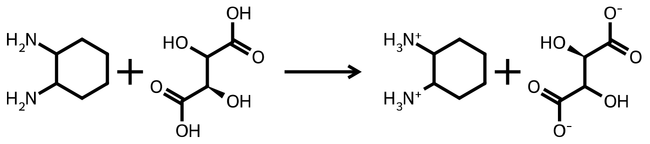
	Step 2			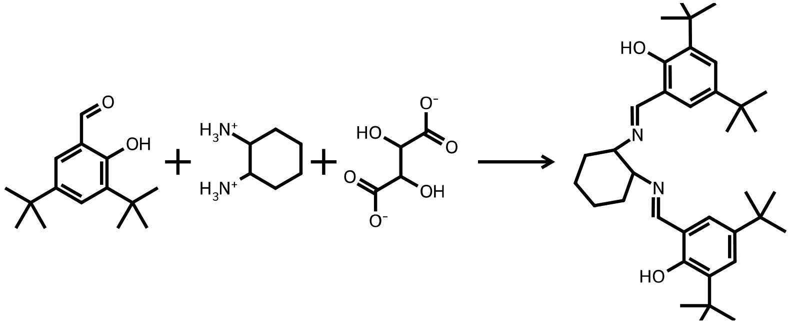
	Step 3			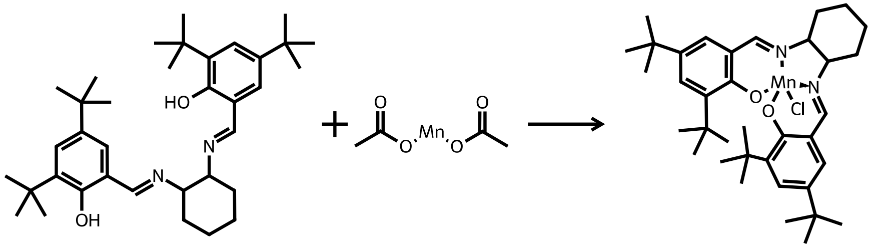
